# Quantifying Delivery of Exercise: Beyond Attendance

**DOI:** 10.1093/geroni/igaf122.3835

**Published:** 2025-12-31

**Authors:** Valerie Shuman, Jessie VanSwearingen, Subashan Perera, Janet Freburger, Kathleen Mangione, Jennifer Brach

**Affiliations:** University of Pittsburgh, PITTSBURGH, Pennsylvania, United States; University of Pittsburgh, Pittsburgh, Pennsylvania, United States; University of Pittsburgh, Pittsburgh, Pennsylvania, United States; University of Pittsburgh, Pittsburgh, Pennsylvania, United States; Arcadia University, Glenside, Pennsylvania, United States; University of Pittsburgh, Pittsburgh, Pennsylvania, United States

## Abstract

Most studies assessing the effectiveness of multi-component exercise programs in older adults report attendance as a proxy for dose without accounting for the extent to which intervention components were completed as intended. We examined associations of component-specific doses of a 12-week, 24-session multi-component walking intervention (including endurance treadmill walking and hip extension/abduction strength training) with 12-week change in gait speed. Dose for each component was quantified as total volume, average intensity, and progression. Participants (N = 118) included community-dwelling older adults aged ≥65 years (77.4 ± 6.3) who reported walking difficulty and had a baseline gait speed between 0.6–1.2 m/s (1.1 +/- 0.2). They attended an average of 21.0 ± 3.7 sessions and walked on the treadmill between 1.0 – 3.4 mph on the treadmill. Strength load progressed by an average of 45.3 ± 38.5 and 29.1 ± 47.0 per session for hip extension and abduction, respectively. Attendance was not associated with improvement. However, higher average treadmill speed, faster progression in treadmill speed, and greater progression of hip strength training loads were each associated with greater gains in gait speed. For example, increasing the treadmill speed by 0.1 mph every 5 sessions was associated with a 0.2 m/s increase in gait speed. The findings illustrate the fallacy of using only attendance as a proxy for dose. A detailed assessment of dose received for each component of a multicomponent exercise program offers valuable insight into which components drive functional improvement. Such findings can inform better care delivery.

